# Solar or UVA-Visible Photocatalytic Ozonation of Water Contaminants

**DOI:** 10.3390/molecules22071177

**Published:** 2017-07-14

**Authors:** Fernando J. Beltrán, Ana Rey

**Affiliations:** Departamento de Ingeniería Química y Química Física, Instituto Universitario de Investigación del Agua, Cambio Climático y Sostenibilidad, Universidad de Extremadura, Av. Elvas s/n, 06006 Badajoz, Spain; anarey@unex.es

**Keywords:** solar photocatalytic oxidation, ozonation, solar photocatalytic ozonation, water contaminants

## Abstract

An incipient advanced oxidation process, solar photocatalytic ozonation (SPO), is reviewed in this paper with the aim of clarifying the importance of this process as a more sustainable water technology to remove priority or emerging contaminants from water. The synergism between ozonation and photocatalytic oxidation is well known to increase the oxidation rate of water contaminants, but this has mainly been studied in photocatalytic ozonation systems with lamps of different radiation wavelength, especially of ultraviolet nature (UVC, UVB, UVA). Nowadays, process sustainability is critical in environmental technologies including water treatment and reuse; the application of SPO systems falls into this category, and contributes to saving energy and water. In this review, we summarized works published on photocatalytic ozonation where the radiation source is the Sun or simulated solar light, specifically, lamps emitting radiation to cover the UVA and visible light spectra. The main aspects of the review include photoreactors used and radiation sources applied, synthesis and characterization of catalysts applied, influence of main process variables (ozone, catalyst, and pollutant concentrations, light intensity), type of water, biodegradability and ecotoxicity, mechanism and kinetics, and finally catalyst activity and stability.

## 1. Introduction

For wastewater to be released into a natural water environment or reused for social or industrial purposes, it first passes through the classical unit operations of a wastewater treatment plant (WWTP). It comes out as apparently clear water, with chemical and biochemical oxygen demand values (COD, BOD) normally below those allowed by law or by official environmental rules. However, such treatments often do not remove organic compounds, called micropollutants, at very low concentrations (μg to ng L^−1^). These contaminants are generally due to human activities relating to agriculture, industries, or simply health and personal care. Thus, compounds such as pesticides, phenols, and pharmaceuticals, among others, are frequently found in the wastewater influent and effluent of WWTPs and in groundwater [1,2,3,4]. Many of these organics have well-defined maximum contaminant levels (MCL) (priority pollutants) [5], but others still do not have MCL and are called emerging contaminants. Thus, phenols and pesticides are priority pollutants while pharmaceuticals and personal care products are emerging contaminants. These compounds are a threat to water quality in different ways. For instance, antibiotics can increase the resistance some microorganisms have. A variety of pollutants can disrupt the endocrine system, causing tumors, sexual changes in animals, etc. [6]. In addition, water scarcity and/or drought increases the need for water reuse and makes necessary the effective elimination of biologically active compounds. Therefore, tertiary treatment methods such as adsorption, membrane, and chemical oxidation processes should be included in WWTPs to address these problems. Adsorption and membrane operations only transfer contaminants from the treated water to a second phase (the adsorbent or the concentrate in the membrane process), while chemical oxidation can eliminate the pollutants.

Chemical oxidation can be accomplished with the use of individual chemicals such as ozone or hydrogen peroxide, or through combinations with other agents such as radiation and/or catalysts. These combinations are usually called advanced oxidation processes (AOPs), since they generate hydroxyl radicals (HO·). The strong oxidation potential of HO∙ can degrade or eliminate essentially all micropollutants except perhalogenated compounds. The rate constants of the reactions between HO∙ radicals and most organic compounds range from about 10^7^ M^−1^ s^−1^ for the most recalcitrant organics (i.e., oxalic acid) up to 10^10^ M^−1^ s^−1^ for the most reactive compounds (i.e., phenol) [7].

**Figure 1:**
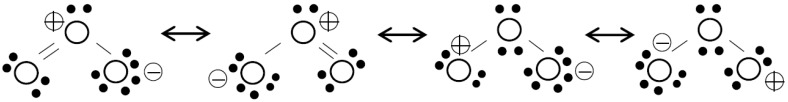
Electronic forms of an ozone molecule.

Because of its high reactivity and potential combination with other agents, ozone forms an important group of AOPs. In fact, ozonation is an AOP alone since ozone may decompose into hydroxyl radicals or upon direct reactions with different organic compounds [8]. The reactivity of ozone is due to its electronic structure (see Figure 1), which has resonance forms with positively and negatively charged oxygen atoms.

**Figure 2:**
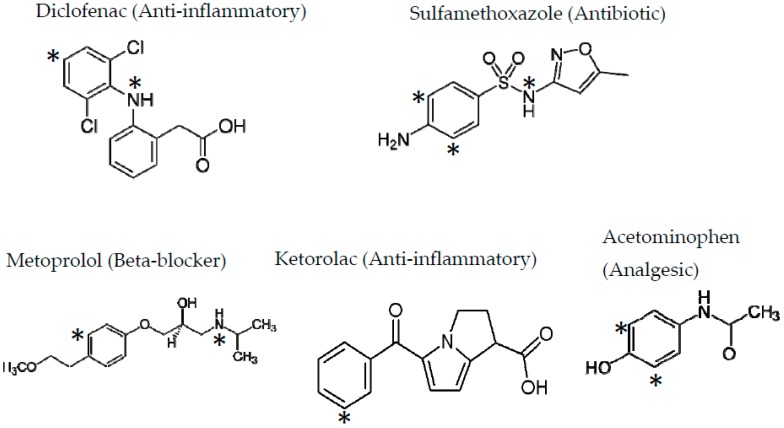
Examples of molecular structures of some pharmaceuticals usually found in urban wastewater. * Possible points of ozone attack.

Ozone, which is an oxidant, reacts very fast with organic compounds with electron-rich systems such as double carbon bonds and aromatic rings. Pharmaceuticals often contain electron-rich functional groups in their structure and are very highly reactive to ozone attack (see Figure 2). The rate constants of the reactions of ozone with these compounds can also be very high, as seen in Table 1.

Consequently, in an ozonation process there are two types of reactions with the organics in water: the so-called direct reactions, and the reactions of hydroxyl radicals formed from the decomposition of ozone. The combinations of ozone with different agents condition the way hydroxyl radicals are formed and their concentration. For example, the peroxone process, that is, the combination between ozone and hydrogen peroxide at neutral pH, is a well-known AOP. In this case, the reaction of ozone with the hydroperoxide ion (the ionic form of hydrogen peroxide) initiates the formation of free radicals in a reaction with a high rate constant (2.8 × 10^6^ M^−1^ s^−1^) [16], while the reaction that initiates the formation of free radicals in ozonation alone has a value of only 70 M^−1^ s^−1^ [17]. This is the reason why ozonation alone is, let us say, a poor AOP because of the low generation rate of free radicals. Other combinations of ozone with catalysts and/or radiation constitute other ozone AOPs where, in different ways [8,18,19,20,21], hydroxyl radicals are formed, as occurs with photocatalytic ozonation.

Photocatalytic ozonation is the combination of ozonation and photocatalytic oxidation, which is the application of radiation upon a semiconductor in the presence of oxygen or air. This process started in 1978, with the first published work dealing with the removal of contaminants in water [22]. Six years before, in 1972, it was reported that UV-irradiation of a TiO_2_ anode produced hydrogen from water using an electrical bias [23]. Photocatalytic oxidation takes place when photons, with an energy equal to or higher than the band gap energy of the catalyst (or semiconductor) used, excite electrons from the highest occupied molecular orbital (HOMO) or the valence band to be transferred to the lowest unoccupied molecular orbital (LUMO) or conduction band of the catalyst. In this way, two charge carriers occur: an oxidizing point in the HOMO or hole, and electrons able to trigger reduction reactions in the LUMO [24,25,26,27]. The main problem with this process is that the electron-hole pair recombination inhibits the oxidation-reduction steps. Both oxidizing holes and electrons in the presence of adsorbed water and/or contaminants give rise to the formation of hydroxyl radicals or molecular oxidizing substances such as hydrogen peroxide [28].

From the beginning, there has been a great research interest in both ozonation and photocatalytic oxidation processes. In fact, a search in the Web of Science database from 2000 to the present day, with the keywords ozonation and water or photocatalytic oxidation and water, produces 5656 and 10,434 papers published on the two processes, respectively. Furthermore, their main characteristics, possible applications, and experimental and practical results (with regard to ozonation) have been the subject of different reviews [21,25,26,27,28,29,30,31,32,33,34,35].

**Figure 3:**
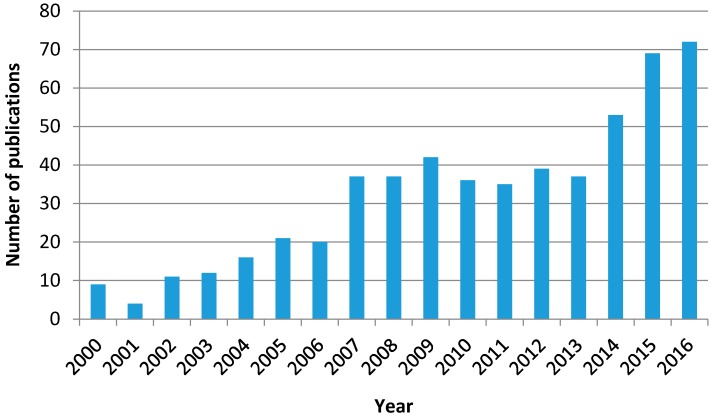
Evolution of the number of publications on water photocatalytic ozonation in the period 2000–2016.

Given the fact that ozone is a stronger oxidant than oxygen, the simultaneous application of ozone, radiation, and a catalyst with the abovementioned features has led to a new AOP called photocatalytic ozonation. Thus, Figure 3 shows the evolution of the numbers of publications on photocatalytic ozonation that have appeared in scientific literature from 2000 to 2016 according to the Web of Science database.

Although the number is far lower than the corresponding figures of ozonation and photocatalytic oxidation as individual processes, the trend of publication is clearly increasing with time. In spite of its short history, this subject has already been the focus of some reviews where the characteristics, operating variables, mechanism, and kinetics of the process are dealt with from the works published so far [36,37,38,39].

In this work, however, the subject under study is not photocatalytic ozonation, but the logical consequence of this AOP to make it more environmentally sustainable: solar photocatalytic ozonation (SPO), or the use of solar or simulated solar radiation in the photocatalytic ozonation process. Thus, the main features of this process are given below with emphasis on the appropriateness of this emerging AOP, the catalysts, the organics treated, the kinetics and the mechanism, etc.

## 2. The Solar Photocatalytic Ozonation Process

Photocatalytic ozonation with UVC or UVA lamps yields significant reductions of many organics and total organic carbon (TOC) of the contaminated water in a very reduced reaction time (minutes for organics and a few hours for TOC) if compared to ozone-free photocatalytic oxidation, but the process is expensive because both the generation of ozone and lamp functioning require electrical energy which can be significant in many cases. Conversely, solar photocatalytic ozonation only requires energy to produce ozone; moreover, this energy will eventually be able to be produced from the Sun with a suitable photovoltaic or similar system able to generate electrical energy from solar energy. Oyama et al. [40], for example, studied the removal of 2,4-dichlorophenoxyacetic acid, bisphenol A and two surfactant compounds with SPO, and the electric power for the reactor system, pumps, ozone generator, radiometer, and computer used was supplied entirely by a solar cell battery. The Sun can be a never-ending natural energy resource. The average incident solar energy on the Earth’s surface is about 240 W m^−2^, a part of which (about 5%) corresponds to UVA radiation with wavelengths from 290 to 400 nm, (5% UVB and 95% UVB) However, total solar energy (290–800 nm) would be approximately three thousand millions of MW, which is equivalent to about 15 million 2000 MW Nuclear Power Plants or about 600 million of 50 MW thermosolar or photovoltaic plants. Thus, solar energy could be the solution for environmental sustainable processes such as the photocatalytic degradation of water contaminants.

It might be said that works on SPO started in 2002, with an incipient study to remove *p*-nitrophenol and observe the effects of phosphates [41]. However, the first work in which SPO was applied as a main AOP was for the removal of 2,4-dichlorophenol with ozone or hydrogen peroxide, with Fe(III) as catalyst and different radiation sources from UVA blue fluorescent lamps emitting radiation between 300 and 420 nm as well as the Sun itself [42]. The main processes studied were solar photo-Fenton and SPO. In this paper, the greatest TOC removal achieved by SPO (approximately 90% with 20 μEinstein s^−1^) was a clear indication of the high efficiency of this process compared to others, such as solar photo-Fenton, which only allowed 10% mineralization. A search on the Web of Science database from 2000 to 2017 produced 53 papers when the keywords solar photocatalytic ozonation and water were used. However, a closer look at these papers reveals that only 18 of them specifically study the SPO process. In addition, we found a further eight papers on SPO that did not appear in the search. Accordingly, it can be said that SPO is an emerging AOP since, so far, as many as five papers have been published in only one year. In Table 2, the main features of these papers regarding catalysts, organics treated, radiation sources, concentrations of ozone, types of AOPs, etc., are presented. In the following sections, explanations of the results obtained in these works are given.

### 2.1. Radiation Sources and Photoreactors Used

SPO processes are fed with solar radiation that covers the range between 290 to 800 nm, mainly UVA and visible zones of the solar spectrum. This means that radiation sources are limited to the Sun itself and lamps simulating this radiation wavelength range. The exciting gas of these lamps is in many cases Xe, but blue fluorescent lamps emitting from 300 to 420 nm have also been used [42], in this latter case to study part of the visible wavelength range. Also, Xe lamps emit over a wider range, but radiations below 300 nm are avoided with the use of filters. Also, in some studies, filters are used to allow specific radiation wavelength ranges, 320–800 nm or 390–800 nm, to be emitted from Xe lamps in order to reach the water to be treated. For example, Quiñones et al. [50] used a 1500 W air-cooled Xe arc lamp with the emission restricted to wavelengths over 300 nm because of the presence of quartz and glass cut-off filters. The irradiation intensity was kept at 550 Wm^−2^ and the temperature of the system was maintained between 25 and 40 °C throughout the experiments. To cut off all the wavelengths below 390 nm and 320 nm, films of flexible polyester from Edmund Optics and Unipapel, respectively, were used. In some cases, halide lamps were used. For experiments in the laboratory, Shin et al., [48], for example, used three Osram metal halide lamps of different powers (100, 250, and 400 W), which were placed 60 cm above a compound parabolic collector (CPC) reactor (see below) at the top of the chamber as an artificial solar light. According to the authors, the irradiation wavelengths of the metal halide lamps (300–800 nm) and solar light (λ > 300 nm) were similar in terms of the light spectrum. In another study, Mano et al. [53] used a 300 W Xe lamp with an IR cut-off filter; the incident light power inside the vessel, measured by a radiometer, was about 200 mW in the wavelength range from 360 to 470 nm. An additional cut-off filter (λ > 410 nm) was used for visible light irradiation. Also, Liao et al. [49] used a 500 W Xe lamp jacketed by a quartz thimble filled with flowing and thermostated aqueous NaNO_2_ solution (1 M) between the lamp and the reaction chamber as a filter to block UV light (λ < 400 nm). High or medium-pressure Hg lamps have also been used. Mecha et al. [62], for instance, used a Heraeus TQ 150 W medium-pressure Hg lamp that was surrounded by a quartz cooling water jacket and 70 mW cm^−2^ of light intensity. The lamp emission spectrum had peaks at 253.7, 313 and 366 nm in the UV range and 436, 546 and 578 nm in the visible range. Results when this lamp was used could not exactly be classified as due to SPO because of the UVC 254 nm radiation peak; however, this work also used sunlight.

Regarding photoreactors, two main installations have been used, depending on the radiation source. Thus, when lamps, mainly Xe lamps emitting radiation with wavelengths higher than 300 nm, were used, photoreactors were made of glass in cylindrical or spherical shapes where the water containing the catalyst and the organics to be treated was continuously charged or recirculated as it was pumped from a reservoir or a tank. The photoreactor, if it is of semi-batch type, has inlets for ozone feeding and sample taking as well as an outlet for the gas to exit. In many cases, the photoreactor was inside a box or solar simulator also containing the lamp. In all cases, the experimental installations were supplied with connections to the ozone analyzer and ozone destruction units. The second important photoreactor, used to receive natural solar radiation, was in most cases the so-called CPC photoreactor or compound parabolic collector. The CPC photoreactor consists of a number of borosilicate glass tubes connected in series situated above an anodized aluminum parabolic platform or collector oriented to the south and tilted at an angle equal to the latitude of the place where the reactions are carried out. For example, Quiñones et al. [56] used a CPC photoreactor supplied with four borosilicate glass tubes (32 mm external diameter, 1.4 mm thickness, 750 mm length), anodized aluminum reflectors tilted at 45 degrees (the latitude of the place was in this case 38°52′) and inlets and outlets for the gases. The total collector surface was 0.25 m^2^ and the illuminated volume was 1.8 L. In these reactors, a radiometer is usually included to measure the instantaneous and accumulated UV light absorbed. Quiñones et al. [56] used a broadband UV radiometer (290–370 nm) tilted at the same angle as the CPC. In order to introduce the oxidizing gas (ozone-oxygen or ozone-air), at the edge of some of the tubes there were porous plates connected to the gas circuit. The system is usually completed with a reservoir tank for the water that is recirculated through the CPC at a turbulent regime with the aid of a pump.

### 2.2. Catalysts Used

Catalysts or semiconductors used in SPO can be classified into three different types: metal oxides, metal-doped metal oxides, and composites of different materials. All these catalysts are charged to the reacting systems as solids, usually of nanometer size, so heterogeneous photocatalytic oxidation develops. Metal or metal oxide-supported catalysts on solid structures (glass film, Raschig rings, granular activated carbon, etc.) have not yet been used for solar photocatalytic ozonation systems with the exception of the work of Oyama et al. [45] in which, in addition to the conventional TiO_2_ suspension form, the authors also used TiO_2_ coated on glass. Additionally, in some cases, Fe(III) as a homogeneous catalyst was used. In this case, Fe(III) through the formation of aqua complexes, mainly Fe(OH)^2+^, undergoes photolysis with radiation (λ > 300 nm) to give hydroxyl radicals and Fe(II) [56]. Due to the presence of ozone, hydrogen peroxide is also formed from ozone direct reactions [66] or simply by ozone decomposition [17,67]. Then, Fe(II) is reconverted to Fe(III) by reacting with hydrogen peroxide, which is the Fenton reaction [68]. Thus, the addition of Fe(III) as a catalyst in solar photocatalysis allows different ways of hydroxyl radical formation, including those of Fenton and photo-Fenton processes.

Apart from the use of Fe(III), the rest of the works already published (see Table 2) used a solid as a main catalyst or semiconductor. From the three different types of solid catalysts indicated above, metal oxides are the most frequently used, and of these TiO_2_, due to its high catalytic capacity, is the main catalyst used since it first appeared in 1978 [22]. Although TiO_2_ has been prepared and applied in different works, the commercial P25 TiO_2_ from Degussa is actually the most frequently used catalyst. This is due to its low cost, stability (it does not leach into the water), and activity, especially with UV radiation. P25 TiO_2_ contains around 75% anatase and 25% rutile crystalline phases which confer a highly active character [28]. Consequently, this catalyst is often used as a model to compare the activity of new catalysts. These comparisons of catalyst performance can be seen in many of the works quoted in Table 2. Other metal oxide catalysts used in SPO are Bi_2_O_3_, Nb_2_O_5_, SnO_2_, WO_3_, Fe_2_O_3_, In_2_O_3_, Fe_3_O_4_, and CeO_2_ [44,53,55,59,64] which have a band gap energy lower than that of TiO_2_ and hence, a priori, they can be excited with visible light (λ > 400 nm). In addition, the conduction band redox potentials of these catalysts are more negative than that of ozone, so ozone can trap electrons from their conduction bands, generate hydroxyl radicals, and avoid electron-hole recombination [36]. WO_3_ and TiO_2_ have also been applied as a double metal oxide catalyst, as is seen in the work of Rey et al. [52], in which P25 TiO_2_ and TiO_2_ nanotubes were coated with nanosized WO_3_ particles. These catalysts present lower band gap energy due to the tungsten oxide and the charge transfer between photogenerated electrons from the conduction band of TiO_2_ to the WO_3_ conduction band, and holes transfer from the valence band of WO_3_ to the TiO_2_ valence band is favored. Another group of catalysts used in SPO are metal-doped TiO_2_ materials. Of these, Cu, Ag, Fe, Mn, Zr, Ce, and B have so far been used. An Au-Bi_2_O_3_ catalyst was also prepared and applied to remove the Orange II dye in the work of Anandan et al. [44]. One of the possible ways TiO_2_ can be active with visible light is by doping it with different metals. For instance, oxygen atoms in the TiO_2_ lattice can be substituted by B atoms by mixing the p orbital of B with O_2_ p orbitals, narrowing the band gap and thus shifting the optical response into the visible range [69]. Boron can also be located in interstitial positions of the TiO_2_ lattice, leading to the partial reduction of Ti(IV) to Ti(III), which can act as an electron trap enhancing the photocatalytic activity of TiO_2_ [69,70]. A third group of catalysts are composites formed by the combination of metal oxides (mainly TiO_2_) and activated carbon, carbon nanotubes, and some other metal oxides. Examples of composites are Fe_2_O_3_/TiO_2_/activated carbon (FeTiC), WO_3_/TiO_2_, and gC_3_N_4_ or gC_3_N_4_-rGO [49,50,52,60,61], where rGO stands for reduced graphene oxide. The main components are the magnetite crystalline phase that confers a magnetic moment causing the FeTiC catalyst to be easily separated from water, enhanced adsorption capacity, and the lower band gap of Ti-W composites and the narrower band gap energy of WO_3_/TiO_2_ and gC_3_N_4_ catalysts, especially when reduced graphene oxide is present.

### 2.3. Catalyst Synthesis and Characterization

Both the synthesis and characterization of catalysts are important parts of SPO research. Thus, the ways these catalysts have been prepared and characterized are presented in this section. Regarding the synthesis or preparation, the sol-gel method has so far been the most-used method in SPO catalysts. A clear example of this was the TiFeC magnetic composite prepared by Rey et al. [47]. The catalyst was prepared in three steps: first a meso-microporous activated carbon was impregnated with an ethanol ferric nitrate solution. Once iron nitrate was adsorbed, it was dried and subsequently impregnated with ethylene glycol. This was then heated in an oven, allowed to cool, and finally milled into power. Secondly, a sol-gel of titania was prepared from titanium (IV) butoxide diluted in isopropanol. Finally, magnetic activated carbon (FeC) was dispersed in the titania sol and subjected to ultrasonic, evaporation, washing, and drying procedures. A similar but more simple procedure was used to prepare metal doped-TiO_2_. For example, Mecha et al. [62] prepared Cu-, Ag-, and Fe-doped TiO_2_ via sol-gel methods from Ti_3_Cl and metal nitrates, while Quiñones et al. [58] prepared a B-TiO_2_ catalyst from a sol gel where the principal precursors were boric acid in anhydrous ethanol and titanium butoxide. In another study [63], TiO_2_ was prepared from a sol formed from titanium (IV) butoxide, isopropanol, and ultrapure water acidified with HNO_3_. In this work, composites of TiO_2_ and multiwalled carbon nanotubes (MWCNT) were also synthesized from the TiO_2_ sol dispersed with an amount of MWCNT under sonication. Finally, drying and washing procedures were carried out to obtain the TiO_2_/MWCNT composite with TiO_2_ percentages of about 70–80%. Graphitic carbon nitride (g-C_3_N_4_) has also been obtained [49] through a sol-gel method. In this case, the starting material was powdered urea that was heated, washed with ethanol and water, filtrated, and dried. Apart from the sol-gel method, the hydrothermal method was applied in some of the works depicted in Table 2. With this method, Rey et al. [52,55,65] prepared TiO_2_ nanotubes, WO_3_ catalysts, and nanocubes (NC) and nanorods (NR) of CeO_2_. In this latter case, the procedure basically consisted of heating an alkaline aqueous Ce(NO_3_)_3_ solution in an autoclave for a given time at 100 °C for nanorods or at 180 °C for nanocubes. After the hydrothermal treatment, the autoclave was cooled down to room temperature and then the precipitates were separated by centrifugation, washed sequentially with water and ethanol, and dried overnight. A different procedure was used by Mano et al. [53], who carried out a solid-state reaction with Bi(NO_3_) × 35 H_2_O and NH_4_VO_3_ to prepare their BiVO_4_ catalyst (see also [71]). Then, the final product was dried and heated.

The characterization of synthesized catalysts is essential to understand the results of any catalytic process and/or confirm the presence of some metals in any metal-supported catalyst. Thus, works on SPO where new catalysts were prepared also show a section dedicated to catalyst characterization. The main methodologies applied for SPO catalysts are N_2_ adsorption-desorption to establish the specific surface area of the catalyst (SBET) and pore volume (micro and mesoporosities); X-ray diffraction (XRD) to determine the crystalline phases present in the catalyst and the crystalline size; scanning and transmission electron microscopy (SEM, TEM) to obtain photographical information of catalyst samples below 10 or 1 nm diameter, respectively; associated technique energy dispersive X-ray spectroscopy (EDX) to verify the distribution of metal ions in the catalyst particles; X-ray photoelectron spectroscopy (XPS) to provide information about the chemical composition of the catalyst surface; and Fourier Transform-Infrared spectroscopy (FTIR), usually in the wavenumber range between 200 and 4000 cm^−1^, to supply data about the catalyst surface, the presence of functional groups, and interactions between adsorbate and adsorbent. Raman spectroscopy, based on the Raman shift, is also useful for structural characterization, presence of defects, or different crystal size effects in the catalysts. In addition, other important techniques used are: diffuse reflectance UV-Vis spectroscopy (DR-UV-Vis) to determine absorption of radiation in the UV-visible wavelength range of the catalyst, and its band gap energy for potential activation of the catalyst with UVA and visible radiation; inductively coupled plasma to measure metal contents (inductively coupled plasma-mass spectroscopy (ICP-MS) or inductively coupled plasma-optical emission spectroscopy (ICP-OES)); and, for magnetic catalysts, the use of superconducting quantum interference device (SQUID) magnetometry to measure the magnetic moment. In Table 2, the different techniques used in SPO works are listed.

Combinations of these techniques are needed to justify the results obtained. For example, the metal (Fe, Ag or Cu)-doped TiO_2_ catalysts prepared by Mecha et al. [62] were characterized by the combination of specific surface area and DR-UV-vis results to explain the best results obtained with the Fe-TiO_2_ catalyst which presented the highest SBET and red shift of the band edge absorption, with significant tail absorption at 530 nm. In Rey et al. [47], XRD analysis showed the presence of anatase, magnetite, and maghemite crystalline phases in their FeTiC composite. The catalyst was easily separated from water with a magnet and its superparamagnetic behavior was confirmed through SQUID magnetometry. In this work, a very uniform distribution of Fe on the composite was also observed by SEM and EDX and pore volume and specific surface area were measured for the three solids used, activated carbon (AC), Fe-impregnated activated carbon (FeC), and the final composite, FeTiC. The results showed the decrease of micropore volume after impregnation (from AC to FeC) and catalyst preparation (from FeC to FeTiC), which justifies the loss of specific interfacial area from 640 to 552 and finally to 331 m^2^ g^−1^, respectively. FTIR, on the other hand, allowed the justification of some functional surface groups belonging to phenols or TiO_2_-OH bonds, quinones and other carbonyl groups and aromatic structures, amongst others. Mano et al. [53] used different metal oxide catalysts of specific interfacial areas ranging between 2.1 and 54 m^2^ g^−1^. DRUV-vis showed visible light absorption properties for WO_3_, Fe_2_O_3_, In_2_O_3_, Bi_2_O_3_, and BiVO_4_ catalysts used, but not for Nb_2_O_5_ and SnO_2_, although some activity of these latter catalysts was attributed to photosensitizing effects. In another work by Yin et al. [61], graphitic carbon nitride linked to a reduced graphene oxide (g-C_3_N_4_–rGO) composite was characterized. TEM analysis showed that the rGO sheet was sandwiched between g-C_3_N_4_ through the polymerization of melamine molecules. On the other hand, through DRUV-vis, Yin et al. [61] reported a red shift to a longer wavelength in the absorption band edge of g-C_3_N_4_–rGO composite, probably due to the presence of rGO in the g-C_3_N_4_–rGO composite, which exhibits a stronger broad background absorption in the visible-light region. This brings about band-gap narrowing and enhanced visible light utilization. While doping TiO_2_ with boron, Quiñones et al. [58] noticed an SBET increase compared to the B-free TiO_2_ catalyst (68 to 125 m^2^ g^−1^) effect attributed to the lower crystal size of the anatase phase in the B-TiO_2_ catalysts. B/Ti (atomic ratio) proportion was measured through both XPS (surface data) and ICP (bulk data). The authors suggest that most of B is located on the surface of TiO_2_ during the sol–gel synthesis. Sassolite boron structure (H_3_BO_3_) and anatase were crystalline forms identified by XRD, revealing that the crystal size decreases with the increasing B content, an effect attributed to the restrained TiO_2_ crystal growing due to the existence of a large amount of boron. XPS confirmed the formation of Ti–O–B structures (interstitial B). XRD patterns of MWCNT, MWCNT-TiO_2_, TiO_2_-P25, and TiO_2_ prepared samples were obtained in a study by Alvarez et al. [63]. According to the results shown, the pristine MWCNTs had a graphite-like structure and only anatase crystalline form was present in TiO_2_ catalysts. Average crystallite sizes were found for TiO_2_ particles in MWCNT-TiO_2_, TiO_2_-P25, and TiO_2_ samples, respectively. TEM images show that MWCNTs tend to aggregate together as bundles with sorption sites including the inner and outer surface of individual tubes, interstitial channels between nanotubes, and external groove sites. Also, heterogeneous, non-uniform coating of MWCNTs by TiO_2_ particles was observed showing both bare MWCNTs and random agglomeration of TiO_2_ particles on MWCNTs surface. Finally, Mena et al. [64] showed TEM images of a size distribution between 25 and 100 nm for CeO_2_ nanocubes (NC), and a thickness of approximately 7.2 nm and lengths between 40 and 200 nm for CeO_2_ nanorods (NR). The application of XRD allowed pure CeO_2_ cubic phase (fluorite structure) to be identified in both types of catalysts. The content of Ce(III) was higher in CeO_2_-NR as observed by XPS and from DRUV-Vis band gap energies of 3.32 eV for CeO_2_-NC and 3.07 eV for CeO_2_-NR were measured, with the result that this latter catalyst was very efficient when visible radiation was used during photocatalytic ozonation.

### 2.4. Organics Treated

The finding of many organic compounds of a pharmaceutical origin in urban WWTPs has given rise to the application of SPO and other AOPs for the study of the removal of these from water. These compounds, also called emerging contaminants (ECs), so-called because they do not yet have maximum contaminant levels assigned, are the most studied ones in SPO works both in ultrapure or urban wastewater. ECs can lead to a number of risks, such as sterility, feminization of aquatic organisms, and bacterial resistance [72,73], and as such they have to be removed from waters. In most of the papers (see Table 2), concentration of these compounds is some orders of magnitude higher than those found in actual wastewater, ranging from some mg L^−1^ to μg L^−1^, when in reality they usually are at ng L^−1^. There is a double reason for this. First, analytical laboratory equipment to measure ng L^−1^ concentrations is very expensive, and also inefficient, given the high number of analyses required (for instance, to perform a kinetic study). Second, the SPO works in many cases aim to check the activity of new catalysts so, in these cases, the main objective is not really the removal of a given contaminant but the performance of a catalyst. In addition to pharmaceutical compounds, other important groups studied in SPO works are pesticides (see Table 2), which are also recalcitrant to biological and physicochemical processes applied in classical WWTPs. Water from agriculture, which is the industrial activity with the highest water consumption (about 70% of the world’s accessible freshwater [74]) may contain a high number of organic pesticides and fertilizers which are eventually dispersed in aqueous environments by runoff or leaching. Finally, phenolic compounds, such as phenol itself or bisphenol A, a plasticizer compound that has been found in many wastewaters [75], and oxalic acid, because its oxidation directly leads to mineralization [76], are also model compounds in SPO works.

### 2.5. Influence of Main Variables

Variables studied in SPO works are the nature of the catalyst, including here the synthesis procedure for a given catalyst, the concentrations of organics and oxidants such as ozone itself, the pH of water, and the intensity and wavelength range of radiation applied. Although this review deals with solar radiation, the use of filters (for instance when Xe lamps are used), permit the study of different wavelength range effects from λ > 290 nm. However, the most studied variable in SPO works is the comparison with other AOPs which could be called blank AOPs, such as single ozonation and ozone-free solar photocatalytic oxidation or solar ozone photolysis. In fact, comparison of these AOPs with SPO results is a necessary step before proceeding to the influence of other variables. It is the first step to know whether the catalyst applied together with ozone and light is worth being investigated to remove water contaminants. Examples of this comparison can be seen in most of the works in Table 2. The normal way to make the comparison is by determining the concentration of the organics and total organic carbon with reaction time, the latter is known as mineralization. In many cases, there are few differences between the organic removal rates observed during the different ozone processes applied (ozonation, solar ozone photolysis, catalytic ozonation, and solar photocatalytic ozonation) because the organics studied react very fast with ozone through their direct reactions so that there is no need to combine ozone with any other agent (catalyst and/or light). A clear example of these results is given in the study of Márquez et al. [51] in which a mixture of ECs including atenolol, ofloxacin, hydrochlorotiazide, and trimetropim is treated. Similar results, that is, scarce differences between ozone processes for the removal of a mixture of six ECs were also found in the works of Quiñones et al. [56,57], both in ultrapure water and urban wastewater. However, the fact that differences between reaction rates of organics are independent of the ozone process applied is not only due to the fast ozone-organic compound direct reaction rates, as can be seen in another study by Quiñones et al. [58]. Here, a mixture of four pesticides with rate constant values of their direct reactions with ozone lower than 400 M^−1^ s^−1^ was treated. As in the preceding case, no differences were observed in the reaction rates for different ozone processes applied, but now the contribution of HO radicals cannot be disregarded, especially in the cases of diuron and t-buthylazine which react slowly with ozone (the rate constants of direct ozone reactions are 3.7 and 20 M^−1^ s^−1^ for diuron and, terbuthylazine, respectively, [58]). It is likely that direct reactions with some of the organics present in water that yield hydrogen peroxide will trigger the formation of hydroxyl radicals and facilitate the removal of other more recalcitrant compounds such as terbuthylazine,. In fact, when this herbicide has been treated alone [63], differences between ozonation alone and solar photocatalytic ozonation are important. Thus, the complete removal of terbuthylazine, when treated alone, was observed after 30 min in a solar photocatalytic (P25-TiO_2_) ozonation run, while at this reaction time ozonation only yielded about 63% removal. With other compounds, for example, the insect repellent DEET [55,64,65] or oxalic acid [49], SPO gives much better results than ozonation alone, which is a clear consequence of the recalcitrant character of these compounds towards direct ozonation (very low ozone reaction direct rate constants: <10 M^−1^ s^−1^), meaning that hydroxyl radicals are the only means of oxidation [65]. Another important fact to highlight when comparing AOP processes to remove organics is that, in all cases, SPO is a better option than ozone-free photocatalytic oxidation, since much more time is always needed in this latter process to remove the organics. To give an example of this, the removal of dichloroacetonitrile with SPO and ozone-free solar photocatalytic oxidation with P25 TiO_2_ as a catalyst, after 4 h of reaction, was 90% and 20%, respectively [48]. Significant differences can also be observed with other compounds (see Table 2). Another common result of all SPO works is related to TOC removal. In this case, regardless of whether the compounds studied are alone or in a mixture with other compounds, TOC removal or mineralization is always much better with SPO than with any of the other AOPs examined, that is, blank AOPs such as ozonation alone or combined with the catalyst or UVA-visible or solar radiation. This can be observed in any of the studies listed in Table 2.

The effect of catalyst nature is another important variable tested in SPO works. In an attempt to benefit from the visible zone of the solar electromagnetic spectrum, many catalysts are prepared to be active with radiation wavelengths higher than 400 nm. The TiO_2_ catalyst, due to its high band gap (3.2 eV), is only active with UV radiation, though the presence of defects in the crystalline structure shifts its absorption capacity to the visible light radiation wavelength in some cases. In order to activate this situation, metal doping of TiO_2_, composites of TiO_2_, or other visible active catalysts have been used. In these cases, SPO studies show results with Xe lamps and filters that cut wavelength radiation range. For instance, Quiñones et al. [50] studied the removal of metoprolol (MTP) with a FeTiC composite and a Xe lamp so that the use of filters allowed the passing of three radiation wavelength ranges: 300 to 800 nm, 320 to 800 nm, and 390 to 800 nm. Thus, during ozone photolysis, after 5 h of reaction, mineralization was about 60% with irradiated light between 300 to 800 nm but was reduced to 40% with radiation lights from the other two wavelength ranges. This means that the main photolysis of ozone to yield hydroxyl radicals happens between 300 to 320 nm, which is in accordance with ozone quantum yields [77]. In the presence of the best FeTiC composite catalyst, mineralization percentages were about 92%, 90%, and 60%, for radiation lights of 300 to 800 nm, 320 to 800 nm, and 390 to 800 nm, respectively, which confirm the 300 to 320 nm interval as the most efficient for TOC removal. In another paper [61] in which a g-C_3_N_4_–rGO composite was prepared and used as a catalyst with a NaNO_2_ aqueous solution filter, the authors reported that the contribution of UV light (λ < 400 nm) and visible light (λ > 400 nm) to the degradation of oxalic acid by O_3_/UV-Vis/g-C_3_N_4_–rGO system (see Table 2) was 33.1% and 66.9%, respectively. Other visible light active catalysts prepared were Bi_2_O_3_, WO_3_, or metal doping TiO_2_ (with metal = Cu, Mn, Fe, Zr, etc.) [44,46,54,55]. For instance, in the study by Feng et al. [46] the M-TiO_2_ catalysts showed extended absorption spectra into the visible-light region. Ag-TiO_2_, Cu-TiO_2_, Ce-TiO_2_, and Fe-TiO_2_ showed a relatively small absorption region between 400 and 580 nm, while Mn-TiO_2_ and Zr-TiO_2_ exhibited substantial and broad absorption shoulders of up to 700 nm.

Another variable studied is the effect of changes in some steps of the synthesis procedure. For example, Quiñones et al. [58], while preparing a boron-doped TiO_2_ catalyst, changed the amount of B containing precursor (boric acid) to have different mass B percentages in the final catalyst. They observed that the catalyst with the highest amount of B leads to the highest removal rate (the maximum B percentage was 12%, though subsequently they observed that some B had leached). According to the authors, the probable reason is that B tends to lose its three valence electrons which are transferred to the 3d orbitals of lattice Ti ions yielding Ti(III), diminishing the electron-hole recombination. In the FeTiC/solar/O_3_ process [50], the synthesis procedure included changes that affected the amount of Fe incorporated into the catalyst composite, which obviously produced changes in the saturation magnetization of catalysts and in TiO_2_ content. The increasing presence of Fe affected the specific surface area that became lower but with more TiO_2_. These two effects changed the amount of organic compound adsorbed and reacted on the catalyst surface. Thus, the catalyst with the lowest Fe content had the highest adsorption capacity, while the catalyst with the highest TiO_2_ content presented the highest activity. Calcination time and/or temperature effects were studied for the preparation of WO_3_, which led to different crystalline structure catalysts [54,55]. These works show that monoclinic or orthorhombic forms of WO_3_ were more active for the photocatalytic ozonation process using visible light from λ > 390 nm or λ > 300 nm for the total solar spectrum.

Apart from catalyst and AOP comparison, the influence of other main variables that affect the oxidation rate, that is, pH, organics, ozone and catalyst concentrations, and intensity of radiation, has not been extensively examined in SPO works. In fact, only in some cases [48] has the influence of pH, ozone and catalyst (P25 TiO_2_) concentration, and temperature been studied. In ozone processes, but also in adsorption processes, pH is a fundamental variable and hence its effect on SPO must be optimized. pH therefore affects the charge distribution of the catalyst surface, which will depend on the pH_pzc_ of the catalyst and dissociated species present in water. It also affects ozone decomposition in free radicals and even ozone direct reaction rates with dissociated species [78]. Shin et al. [48] studied the SPO of dichloroacetonitrile (DCA) at pH 3, 6.5 and 10. They observed that DCA self-decomposes at pH 10 while it remains unaltered at the other two pH values. In the SPO runs, they observed a positive increase in reaction rate with the increasing pH. At pH 10, the reaction rate was probably due to the action of hydroxyl radicals coming from ozone decomposition and photocatalysis. However, the reaction rate at pH 6.5 was also significant and in this case, given the pH_pzc_ of TiO_2_ (6.2–6.6 [79,80]), pH 6.5 is also likely to be the best value for SPO application since, as the authors indicate, there is no need to add any basic or acid substance.

The effect of the concentration of organics on the removal rate during SPO runs has only been studied by Rey et al. [47]. They treated MTP at concentrations of 10 and 50 mg/L and they observed a similar behavior to the other ozone processes were studied, that is, for a given reaction time, an increase in the initial organic compound concentration leads to an increase in removal rate (both for the compound and TOC). The logical consequence of these results is that reaction rates are proportional to the concentration of the organics treated, which usually follow a Langmuir reaction rate equation. Obviously, the complete disappearance of MTP takes more time when the initial concentration is higher. A comparison was also made between the results obtained in solar photolytic ozonation and SPO at different concentrations. These authors noted that at 10 mg L^−1^, concentration differences in the TOC removal rate were not significant, but at 50 mg L^−1^, the removal rate through SPO was much higher than with solar photolytic ozonation. The effect of the initial concentration of organics has been more extensively treated in non-solar photocatalytic ozonation processes with similar results to those presented above [81,82].

With regard to the effect of the catalyst concentration, an increase in this variable leads to an increase in the reaction rate due to the increase in surface, that is, active centers. However, this is observed up to an optimum value above which the reaction rate diminishes. The reason is poor light transference or photon absorption rate through a higher concentrated catalyst suspension. Shin et al. [48] reported an optimum value of 1 g L^−1^ for the SPO removal of DCA, which is similar to others found in some solar photocatalytic oxidation processes [31].

The ozone concentration effect is also positive up to a given value, above which no influence on the reaction rate is observed. This happens in any type of ozone process and is related to the ozone saturation of water [8]. In the study by Shin et al. [48], the optimum ozone dose found was 1.13 g L^−1^ h^−1^. This optimum value is also highly dependent on the nature of the organics in the water, pH, and catalyst concentration because all of these affect the ozone driving force in the water.

Temperature also has an optimum value because of two opposing effects. A temperature increase leads to an increase in reaction rate constants, but at the same time to a decrease in ozone solubility, which are opposing aspects. Thus, in SPO runs of Reference [48], the optimum temperature was 20 °C in the range from 10 to 40 °C.

Finally, the study by Shin et al. [48] is the only one that has analyzed the effect of light intensity on an SPO system. As a rule of thumb, in photocatalytic oxidation processes, reaction rate and light intensity are proportional up to a given value of the latter, above which the reaction rate becomes proportional to the square root of light intensity [31]. Shin et al. [48] varied the light intensity between 4.6 and 33.8 Wm^−2^, and found that about 20 Wm^−2^ was needed to change the proportionality of reaction rate/light intensity, which is in accordance with what has been mentioned above.

In a practical situation, SPO will probably be applied to remove contaminants in a given wastewater, simply for depuration or for recycling. Thus, the effect of the water matrix in SPO also needs to be examined. Marquez et al. [51] studied the removal of ECs in a mixture dissolved in ultrapure water and wastewater from a secondary effluent. These authors observed a decrease in reaction rates in wastewater, but the effect of the water matrix could not be established because they used different EC concentrations in each water type. In fact, there are no studies on SPO where the effect of the water matrix on organic compound or TOC removal rate has been studied with equal organic concentrations. However, the presence of hydroxyl radical inhibitors in wastewater actually makes the SPO process slower than in ultrapure water.

Given that in a real situation, SPO will be an additional process of a biological step (for instance, a secondary treatment of urban wastewater), Gimeno et al. [59] studied the aerobic biological oxidation followed by SPO of a mixture of nine ECs (see Table 2) that were doped in an urban wastewater primary effluent. First, aerobic biological oxidation was applied with a food to a microbial ratio of 0.5, a hydraulic residence time of 7 h, and mixed liquor volatile suspended solids (MLVSS) of 1.5 g L^−1^ with WWTP conventional activated sludge as biomass. The initial concentration of each EC compound was 0.2 mg L^−1^ so that the TOC contribution of ECs was less than 5% of initial TOC of the wastewater primary effluent. The authors observed 50% and 80% COD removals in the presence and absence of ECs, respectively. This indicates that some sort of disturbance to microorganisms present on the activated sludge occurred when ECs were present. The authors highlighted that it could be due to the presence of sulfamethoxazole, an antibiotic which alters microorganism concentration and nature at concentrations higher than 50 μg L^−1^ [83]. After 7 h of biological oxidation, only three ECs (caffeine, acetaminophen, and metoprolol) out of nine present in wastewater underwent some significant reductions (between 40% and 61%), the other ECs only obtaining percentage removals of less than 5%. Gimeno et al. [59] in subsequent experiments applied some AOPs (SPO with TiO_2_, solar photo-Fenton: Fe(III) and magnetite, and single ozonation) to the biologically treated wastewater. With a total accumulated solar energy of 30 kJ L^−1^, more than 80% ECs removal was achieved with SPO TiO_2_. Mineralization was 40%. In the absence of ozone, mineralization was less than 10%. Finally, process efficiency followed this order: SPO magnetite > SPO TiO_2_ > SPO Fe(III) > ozonation >> solar photocatalytic oxidation.

### 2.6. Intermediates, Ozone Consumption, Biodegradability, and Toxicity

In some works, during the course of SPO runs, identification and/or concentration measurement of intermediates was also carried out for two reasons: to check the toxic character of these compounds and/or to establish the mechanism of reactions (see the next section). Because of the simplicity of the analytical procedures, the main intermediates detected were in some cases phenolics (such as total phenolic compounds), saturated carboxylic acids, and inorganic ions. In some other cases, High Performance Liquid Chromatograph-Mass Spectrometry HPLC-MS was used to detect the first intermediates of a molecular structure similar to the initial organic compound studied. Thus, in the work by Quiñones et al. [50], MTP and six intermediates formed were analyzed by HPLC-qTOF. They represented the results as the variation of the area with MTP conversion observing the existence of an MTP conversion (between 40% and 60%), for which the concentration (area) of intermediates reached a maximum value. Mena et al. [65], with similar analytical techniques, detected 22 intermediates of large molecular weight (MW > 160) and a series of low molecular weight saturated carboxylic acids as end products, the latter with ion chromatography, during DEET degradation. Phenolic compound concentration was measured in the works of Marquez et al. [51] and Quiñones et al. [57]. In these studies, SPO application leads to an initial increase in phenolic compound concentration with time to reach a maximum value followed. Then, at more advanced times a continuous decrease in phenolic concentration with time is observed, though this depends on the nature of the starting compounds to be degraded and the catalyst used in the SPO process. Quiñones et al. [57] observed a continuous decrease in phenolic concentration of up to 75% removal with 10 kJ L^−1^ of accumulated Sun energy with SPO TiO_2_ applied to a mixture of seven ECs in a secondary effluent of an urban WWTP. They also observed that ozone-free solar photocatalytic (TiO_2_) oxidation led to an increase of 50% phenolic concentration with 15 kJ L^−1^ of accumulated Sun energy which was reduced at a more advanced reaction time but remained higher than the initial one at the end of the reaction (after 40 kJ L^−1^ of accumulated sun energy). In SPO, the concentration of carboxylic acids was followed in several works, as shown in Table 2. Apart from phenolics, carboxylic acids, and TOC measurements as a way of determining the remaining amount of all intermediates and final products, in many works hydrogen peroxide and dissolved ozone concentration are followed. Hydrogen peroxide is a key compound in ozonation processes since its presence triggers different routes of hydroxyl radical formation (see the later mechanism section). For instance, Mena et al. [64] measured the concentration of hydrogen peroxide with time in experiments of ozonation, ozone photolysis, catalytic ozonation, and SPO (with simulated solar light and CeO_2_ nanocubes) and observed a rapid formation of hydrogen peroxide, much greater in ozonation alone than in the catalytic processes. In fact, the concentration of hydrogen peroxide in SPO was very low, probably not only as a consequence of its reaction with ozone, but also because of its direct photolysis and participation in capturing electrons. However, in Mena et al. [64], using a WO_3_ catalyst, the presence of H_2_O_2_ did not have a beneficial effect as an electron acceptor.

The concentration of dissolved ozone is also followed in SPO works to determine ozone consumption, that is, the amount of ozone needed to remove a given amount of organic carbon measured as TOC. Márquez et al. [51] reported that ozone consumption was much lower in SPO than in other ozone processes to treat four ECs. In ultrapure water, for a 40% TOC removal, they observed 35, 20, 40 and 18 mgO_3_/mgTOC in ozonation alone, catalytic ozonation, solar ozonation, and SPO, respectively, with P25 TiO_2_ as a catalyst. In urban wastewater, also for 40% TOC removal, the figures were 61, 52 and 37 mgO_3_/mgTOC in ozonation alone, solar ozonation, and SPO, respectively. It can be seen then that SPO again leads to the lowest ozone consumption, though it is about twice the value observed in ultrapure water. In wastewater, however, the contribution to TOC of the four ECs studied was negligible.

Biodegradability has been studied as the ratio BOD/COD. As a general rule, it can be said that biodegradability in wastewaters increases with the application of ozone in any ozonation process, SPO included. In particular, Márquez et al. [51] observed that BOD/COD ozone-free photocatalytic ozonation of an urban secondary effluent wastewater remains constant with time but increases from 0.22 (raw wastewater) to 0.78 with SPO. This was the highest value obtained from the ozonation processes applied. Quiñones et al. [57] also reported a 2.5-fold increase in the BOD/COD initial ratio of another urban secondary effluent wastewater, with the homogeneous solar-Fenton photocatalytic ozonation process.

Finally, ecotoxicity is another typical determination in AOP and, in particular, in SPO processes. *Daphnia magna* survival is usually the test performed. Gimeno et al. [59] reported the absence of inhibition in immobilization tests after applying a SPO process to urban wastewater with TiO_2_ or Fe(III) as catalysts. Quiñones et al. [57] studied the toxicity of urban wastewater doped with six ECs (0.2 mg L^−1^ concentration each). The bioassays showed an increase in toxicity after the addition of ECs to the secondary effluent. Thus, the percentage of inhibition increased from 5% (without added ECs) to about 25%. Additionally, changes in sample toxicity were observed during the course of the photocatalytic experiments. As a rule, the toxicity of samples increased at the beginning of the photocatalytic treatment, probably as a consequence of the accumulation of phenolic and other toxic intermediates, and then decreased. This result is in agreement with that reported for the removal of other ECs by ozonation and solar photocatalytic oxidation with TiO_2_ [51,84]. Toxicity removal below 25% inhibition was only observed when a high degree of TOC removal (i.e., mineralization) was achieved. This applies especially for the Fe(III)/O_3_/solar light (pH 3) and Fe(III)/H_2_O_2_/O_3_/solar light (pH 3) photocatalytic ozonation systems.

### 2.7. Mechanism and Kinetics: The Use of Scavengers

The SPO process goes through a complex mechanism of direct reactions between compounds present and oxidants (ozone, generated hydrogen peroxide, oxidizing holes), as well as free radical reactions. This complexity is the result of the combination of the ozonation and photocatalytic oxidation processes. The former, ozonation, implies direct ozone-organic reactions and ozone decomposition reactions due to the appearance of hydrogen peroxide, increase in pH, and ozone photolysis, at least with radiation wavelengths of up to 320 nm. Photocatalytic oxidation involves hydroxyl radical formation from holes in the catalyst valence band and probably from the superoxide ion radical formed from excited electrons of the catalyst conduction band [24,25,26,27]. Detailed explanations of these mechanisms are available elsewhere [56,85]. When ozone, an active semiconductor, and light are simultaneously present to remove some organics in water, some new reactions occur, the main one being the formation of the ozonide ion radical, O_3_•^−^, from the ozone capture of electrons in the catalyst valence band:

(1) $$O_{3}+e^{-}\to O_{3}^{-}•$$

which eventually leads to hydroxyl radicals:

(2) $$O_{3}^{-}•+H^{+}\to HO_{3}•\to O_{2}+HO•$$

In the SPO process, hydroxyl radicals can be formed from the individual mechanisms of ozonation and photocatalytic oxidation processes [17,85] but also from the synergism between these two processes, as it is observed from Reactions (1) and (2) [86]. Thus, in the SPO process, there are some possible oxidizing agents: ozone; hydrogen peroxide, formed in direct ozone reactions with some organics (aromatic compounds, olefines, etc.) [87]; oxidizing holes in the catalyst valence band; singlet oxygen, etc. [88].

The use of scavengers is an effective way to check the importance of the oxidation mechanism or the importance that some oxidants have in a given SPO process. These scavengers react exclusively with some oxidants like hydroxyl or superoxide ion radical or oxidant holes. Liao et al. [49], for example, used tert-butanol and triethanolamine as scavengers of hydroxyl and valence band holes, respectively. With these experiments, the authors observed that after 120 min, the oxalic acid removal efficiency reached 80% without scavengers. However, only 46.2% and 66.5% of oxalic acid was removed with the presence of tert-butanol and triethanolamine separately. This means that both hydroxyl radicals and holes contributed to the degradation of oxalic acid. However, the highest inhibition of removal rate was observed with the addition of tert-butanol, which suggests that HO∙ radicals play a dominant role in the SPO of oxalic acid. Furthermore, in another work [60], benzoquinone was used to scavenge any possible superoxide ion radical that could be formed from conduction band electrons trapped by oxygen:

(3) $$O_{2}+e^{-}\to O_{2}^{-}•$$

Superoxide ion radicals can oxidize organic matter, though the rate constants of their reactions with organics are, in most cases, lower than those of hydroxyl radicals [89,90] and can also react with ozone to yield more ozonide ion radicals:

(4) $$O_{3}+O_{2}^{-}•\to O_{3}^{-}•+O_{2}$$

In fact, Reactions (3) and (4) with Reactions (1) and (2) complete the synergism mechanism between ozonation and photocatalytic oxidation. In this respect, Liao et al. [60] observed a 95% and 37.8% removal of oxalic acid during SPO (with a g-C_3_N_4_ catalyst) in the absence and presence of benzoquinone. The contribution of superoxide ion radicals to remove oxalic acid was also confirmed.

The Langmuir equation, usually applied to follow the kinetics of ozone-free photocatalytic oxidation processes [85], is also used in SPO kinetics. The Langmuir equation is as follows:

(5) $$-r_{M}=\frac{kKC_{M}}{1+KC_{M}}$$

where *k* and *K* are the rate constant of the SPO process and the adsorption equilibrium constant of compound *M*, respectively, and *r_M_* its removal rate. Mecha et al. [62] used simplified Langmuir kinetics reduced to a first order reaction rate because they observed negligible adsorption of the compound they studied, phenol, on the catalyst they prepared (see Table 2). With the obtained apparent pseudo first order rate constants, they calculated the synergy index of SPO, that is, the ratio between SPO rate and the sum of the ozonation and ozone-free photocatalytic oxidation rates:

(6) $$SI=\frac{k_{\mathrm{SPO}}}{k_{O3}+k_{\mathrm{SPoxidatio}}}$$

The authors obtained values of 1.51, 1.03, 1.11 and 1.54 for the SPO processes conducted with TiO_2_, Ag-TiO_2_, Cu-TiO_2_, and Fe-TiO_2_ catalysts, respectively. Thus, bare TiO_2_ and Fe-TiO_2_ were the best catalysts investigated with the highest SPO removal rates.

As mentioned above, the contribution of hydroxyl radical reactions in SPO processes is one of the main ways of oxidation, and the concentration of these free radical species is necessary for kinetic studies. Quiñones et al. [57] applied the hydroxyl radical exposure concept to indirectly measure this concentration. This parameter is a measure of the concentration of hydroxyl radical during a given time in any AOP. It also allows for the determination of the R_CT_, which is the ratio between the time integrating concentrations of hydroxyl radical and ozone in any water which is ozonated. It is based on the kinetics of any AOP process when an ozone non-reacting compound like *p*-chlorobenzoic acid (PCBA) is present in the problem water. PCBA does not react with ozone (the reaction rate constant is lower than 0.15 M^−1^ s^−1^ [91]) but it does react with hydroxyl radicals [7]. After taking into account the mass balance of PCBA in a semi-batch reactor where the SPO process was carried out, the hydroxyl radical exposure for a given reaction time can be obtained from Equation (7) [92]:

(7) $$\int_{0}^{t}C_{HO}\;dt=\frac{ln\frac{C_{\mathrm{PCBA}}}{C_{\mathrm{PCBA}0}}}{k_{HO}}$$

where the left side of Equation (7) is the hydroxyl radical exposure; C_PCBA_, C_PCBA0_ are the concentrations of PCBA at time *t* and at the start of the SPO process, respectively; and *k*_HO_ the rate constant of the hydroxyl radical-PCBA reaction (*k*_OH_ = 5 × 10^9^ M^−1^ s^−1^ [7]). The authors observed that the hydroxyl radical exposure increased with the UV radiation dose for any of the systems tested (see Table 2). For a given radiation dose, the ozone/light systems (pH 3) led to higher HO∙ production than those systems tested in the absence of ozone. The Fe(III)/H_2_O_2_/O_3_/light (pH 3) system can be highlighted as the one which produces the highest HO∙ exposure and, consequently, the highest TOC removal after 5 h of treatment (35%).

Additionally, process ozonation kinetics is studied by first determining the kinetic regime of ozone reactions. This can be established by calculating the Hatta number which, for second order irreversible reactions such as those ozone undergoes with organics in water [8], is defined as follows:

(8) $$Hatta=\frac{\sqrt{k_{O3}D_{O3}C_{M}}}{k_{L}}$$

where *k*_O3_, *D*_O3_, and *k_L_* are the ozone-organic *M* reaction rate constant, the ozone diffusivity in water, and the individual liquid side mass transfer coefficient, respectively. Quiñones et al. [56] studied the SPO kinetics of TOC removal from a mass balance in water in a semi-batch reacting system. In this balance, the different contributions to the TOC removal rates were considered as shown below. It should be noted that TOC is a parameter that depends on the type of water and nature of the compounds present and, hence, rate constants involved will also vary depending on the system. The authors studied two different SPO processes according to the catalyst used: Fe(III) and H_2_O_2_ at pH 3, and TiO_2_ at pH 7. For the complex SPO process at pH 3: Fe(III)/O_3_/H_2_O_2_/solar light, *TOC* mass balance applied was:

(9) $$-\frac{dTOC}{dt}=\left(k_{T2}C_{\mathrm{Fe}(\mathrm{II})}C_{H2O2}+k_{O3}C_{O3}+k_{T3}C_{O3}\right)TOC$$

In Equation (9) the terms of the right-hand side represent the contributions of the Fenton reaction and those of the *TOC* reactions with ozone (direct reactions) and with the fraction of hydroxyl radicals that comes from ozone decomposition initiation-promotion reactions. The apparent rate constant k_T3_ is the result of applying the R_CT_ concept, allowing the concentration of hydroxyl radicals to be expressed as a function of that of ozone [92]. In addition, the authors proposed the following *TOC* balance for the pH 7 SPO process: TiO_2_/O_3_/solar light:

(10) $$-\frac{dTOC}{dt}=\left[k_{UV}+\frac{k_{i}C_{O3}}{1+\Sigma K_{i}C_{i}}+k_{O3}C_{O3}+k_{HO}C_{HO}\right]TOC$$

where the four terms of the right-hand side are the contribution of direct photolysis, photocatalytic ozonation, direct ozone reactions, and reactions of hydroxyl radicals that come from non-photocatalytic ozonation reactions. As can be observed, Langmuir kinetics was considered for the contribution of photocatalysis due to oxidizing holes. Langmuir kinetics was reduced to a first order kinetics since most of the active sites were occupied with water molecules. Also, the direct photolysis of *TOC* was negligible. With these simplifications, Equation (10) eventually leads to a second order equation:

(11) $$-\frac{dTOC}{dt}=k_{T}C_{O3}TOC$$

From experimental data, Quiñones et al. [56] determined all rate constant values of Equations (9) and (11). With the rate constants of ozone involving reactions, they calculated the Hatta number to obtain values of less than 0.3 which confirmed a slow kinetic regime and the validity of the mass balance equation applied [8]. They then determined the percentage contribution of the different pathways for the removal of TOC. For instance, for the Fe(III)/O_3_/H_2_O_2_/Solar light/pH 3 system, they concluded that during the first seconds of reaction, the main contribution to the mineralization process corresponds to the photo-Fenton reaction. Minutes later, the percentage contribution of ozone-involving mechanisms increases due to the ozone accumulation and hydrogen peroxide partial consumption. Therefore, the contribution of ozone processes to mineralization increases with the increasing reaction time, accounting for 72% of mineralization after 5 min of reaction and for 98% after 1 h.

### 2.8. Stability and Activity of Catalysts

There are three important properties of any catalyst: selectivity, activity, and stability. In a few studies on SPO, both activity and stability have been analyzed. For example, regarding stability, Quiñones et al. [58] applied a B-doped TiO_2_ catalyst to analyze the leaching phenomenon, after which catalysts were submitted to water washing at the same conditions as the reaction medium (catalyst concentration and pH). They observed that the boron concentration in the solution reached values as high as 5.5 mg L^−1^ in the case of the highest loading B-doped catalyst (12%), the loss of the total boron being from 46 to 70%. However, the authors observed no further loss of B after using the water-washed catalysts in three consecutive runs, which confirms a certain level of stability and activity. In another work, Rey et al. [47], with a magnetic FeTiC catalyst, observed Ti and Fe leaching at concentrations lower than 15 and 25 μg L^−1^, respectively, in each 2 h SPO run they carried out. Then, after five consecutive cycles using the same catalyst, only 0.04% and 0.6% of the initial Ti and Fe, respectively, were leached into the water with a constant activity for TOC removal and good magnetic separability. Liao et al. [60] also reported high activity and stability of their gC_3_N_4_-rGO catalyst to remove oxalic acid. These authors found a slight decrease in oxalic acid removal percentage (96.7 to 93.8%) after five consecutive runs performed with the same amount of catalyst.

## 3. Conclusions and Future Steps

According to the few papers published on SPO compared to other similar technologies such as photocatalytic oxidation, the main conclusion that can be drawn is that SPO is an incipient but potentially attractive AOP since it projects the image of a green and environmentally sustainable process. Thus, solar energy can supply all the necessary energyneeded for an SPO process, that is, the energy for exciting catalysts (wavelength range depending on the nature of the catalyst), photolysing ozone (mainly in the range 290–320 nm), pump functioning, ozone production and even, in the case of cloudy days, accumulated energy could enable the use of new generation artificial lamps.

So far, SPO processes have been carried out at laboratory (solar simulator box with Xe lamps) and pilot plant scale (solar CPC photoreactors) with different types of catalysts: from the classical TiO_2_ to new composite materials containing some metals, activated carbons, graphene, etc. The new synthesized catalysts that are now being investigated include, in many cases, TiO_2_ as the main active material with other components such as metal or metal oxides (for example, Ag, Cu, Fe, Fe_2_O_3_, WO_3_), or carbon materials (activated carbons, multiwalled carbon nanotubes, etc.) that provide energy states between the valence and conduction bands of TiO_2_. This permits a decrease in TiO_2_ band gap energy and allows it to be active under visible light.

Magnetic catalysts are also used in SPO. In this case, TiO_2_, as the main active catalyst, and magnetite, for supplying magnetic properties, are supported on carbon materials such as activated carbons responsible for a wide surface area of adsorption. These magnetic catalysts are easily separated from water with the aid of a magnetic field.

Sol-gel and hydrothermal methods are still the most frequently used for the synthesis of catalysts for SPO. Condition changes of preparation steps lead to changes in the catalyst activity. Composition, crystalline structure, morphology, textural properties of SPO catalysts are obtained with classical techniques such as XRD, SEM, TEM, N_2_ adsorption and desorption isotherms, etc.

At present, photoreactors are of a cylindrical type: tanks or tubes. Agitated tanks are used inside box simulators supplied, in general, with Xe lamps that emit in the solar radiation range (λ > 290 nm), that which reaches the Earth’s surface. Different filters have been used to simulate radiation wavelength ranges between 290–320 nm, 290–400 nm, and above 400 nm (visible light). Several tubes are in many cases connected in series and placed above parabolic light reflecting structures oriented to the south as compound parabolic collectors (CPC).

A comparison of SPO with other AOPs that individually form the combination of ozone, UVA-visible light, and a catalyst is the first and principal study of papers. In all cases, TOC removal with SPO is much higher than that obtained from individual or blank processes (UVA-visible radiation, ozonation, catalytic ozonation, ozone photolysis, and ozone-free photocatalytic oxidation). The formation of hydrogen peroxide in the SPO process gives rise to an increase in TOC removal or mineralization by reacting with ozone to yield more hydroxyl radicals. However, the individual removal of many compounds is even faster with ozonation alone since in most of these cases direct ozone reactions are the main means of oxidation. This is particularly important in the removal of many pharmaceutical contaminants. The effects of other variables such as concentrations of ozone, organics, and catalysts are similar to those already reported for ozone-free photocatalytic oxidation and ozonation processes.

Ozone consumption results in much lower figures in SPO than in the rest of the ozone processes, and the water matrix strongly affects the organics and TOC removal rates, as has been shown from results in ultrapure and wastewater. Biodegradability, measured as BOD/COD, significantly increases with SPO application, rising by about 250% in an urban secondary wastewater effluent [59]. As a general rule, the toxicity of samples increases at the beginning of the photocatalytic treatment, probably as a consequence of the accumulation of phenolic and other toxic intermediates, and then at more advanced reaction times it decreases.

Reaction (1) or electron ozone capturing that generates the ozonide ion radical, eventually leading to hydroxyl radicals, is the main step of ozone-photocatalytic oxidation synergism. The participation of hydroxyl radicals has been confirmed in SPO works with the use of scavengers aiming at determining whether valence band holes and superoxide ion radicals could also intervene directly as other oxidizing species.

At the concentrations that the organics have in wastewaters (up to a few μg L^−1^), the kinetic regime of ozone reactions is slow, which suggests AOPs and hence SPO as recommended processes to increase organic or TOC removal rates. This is because in slow kinetic regimes both ozone direct reactions and hydroxyl radical reactions compete, and the latter have much higher reaction rate constants. Thus, the application of AOPs that generate high concentration of hydroxyl radicals is recommended to increase the reaction rates.

It is foreseen that in the near future there will be an increase in studies on SPO due to its environmentally sustainable character, especially with new catalysts. The objectives of these studies will most likely be related to visible light activation and catalyst separation from water, two problems that so far have limited the practical application of photocatalytic processes in water treatment.

## Figures and Tables

**Table 1:** Rate constants of ozone reactions with some pharmaceuticals found in urban wastewater *.

Compound	Activity	Rate Constant (M^−1^ s^−1^)	Reference
Sulfamethoxazole	Antibiotic	5.5 × 10^5^	[9]
Diclofenac	Anti-inflammatory	10^5^	[10]
Ketorolac	Analgesic	3.4 × 10^5^	
Acetaminophen	Anti-inflammatory	2.7 × 10^5^	
Metoprolol	Beta-blocker	2.5 × 10^3^	
Carbamacepine	Analgesic	3 × 10^5^	[11]
17α-ethinylestradiol	Hormone	3 × 10^6^	
Tetracycline	Antibiotic	1.9 × 10^6^	[12]
Fenoterol	Breath aider	2.8 × 10^6^	[13]
Gemfibrozil	Lipid regulator	4.9 × 10^5^	
Estriol	Hormone	10^5^	[14]
Lyncomycine	Antibiotic	6.7 × 10^5^	[15]

* At pH 7.

**Table 2:** Research articles published so far on solar or UVA-Visible light photocatalytic ozonation of water contaminants ^a^.

Compounds Treated and Processes Applied	Data on Catalysts, Ozone, and Others	Radiation Source	Photoreactor Type	Observations	Ref.
Effluent from an urban wastewater lagoon. Total coliforms followed solar photolysis photocatalytic oxidation (SPO).	P25 TiO_2_ alone: 2 g L^−1^. Activated carbon (AC) alone: 0.3 g/L. Mixture TiO_2_-AC: 2.3 g L^−1^. Ozone dose or concentration: not given.	Solar light: 10 to 14 h radiation.	Agitated or bubble vessels of 250 mL.	COD removal: 40% with SPO (best result) and AC-TiO_2_. Total coliforms removal: >99.99%. Inorganic ions determined: NO_2_^−^, PO_4_^3−^. Fourier Transform FTR study.	[41]
2,4 dichlorophenol (DCP) at 100 mg L^−1^. H_2_O_2_ concentration: 250 and 75 mg/L in UV/H_2_O_2_ and UV/Fe/H_2_O_2_, respectively. O_3_ + Fe(II) + UV, photo-Fenton, UV + Fe(III), UV + H_2_O_2_, photocatalysis and photolysis.	Two catalysts: 1. Fe(III): 10–30 mg L^−1^ as FeCl_3_. 2. TiO_2_: 0.5 g L^−1^. Ozone: 5.5 g h^−1^.	1. Blue UVA lamp (350 nm); 2.5 μE s^−1^. 2. Solar simulator: 6 μE s^−1^. 3. The Sun: 236 μE s^−1^. 4. Eight 15 W UVA blue fluorescent lamps (300–420 nm): 120 μE s^−1^ (μE s^−1^: μEinstein s^−1^).	1. UV, UV + H_2_O_2_, UV + Fe(II), Photo-Fenton runs in 12 × 13 cm photoreactor. 2. Solar simulator with parabolic mirrors and quartz tube reactor. 3. Three compound parabolic collectors CPC, each with eight tubes in series. 4. One 2 L gas-liquid contactor + tube photoreactor.	Scaling up factors for scaling up to pilot-plant size. Estimation of amount of waste water that could be treated. Analysis: DCP concentration and TOC. DCP degradation is plotted as a function of accumulated photons per liter entering the reactor. Apparent pseudo first order kinetics. Pseudo quantum yield as a function of incident energy.	[42]
2,4-dichlorophenoxyacetic acid, bisphenol A, Sodium butylnaphthalenesulfonate and benzyldodecyldimethyl-ammonium bromide surfactants. 2 mM concentration: 100, 175 and 250 mg L^−1^. Processes: O_3_, O_3_/UV, O_2_/UV/TiO_2_ and O_3_/UV/TiO_2_.	P25 TiO_2_ at 2 g L^−1^.	One 75 W High Pressure Hg lamp (3 mW cm^−2^ at 360 nm) and the Sun.	Twenty Pyrex glass tubular reactors.	TOC measurement.	[40]
Phenol: 0.169 mg L^−1^. Processes: O_3_, O_3_/Vis, O_2_/Vis/cat, O_3_/cat and O_3_/Vis/cat. Ozone process: 0.45 g h^−1^.	Commercial WO_3_ powder and n-TiO_2_: 0.2 g L^−1^.	300 W Xe lamp with a cut-off filter (k > 420 nm).	Pyrex inner-irradiation vessel placed in a water bath set at room temperature.	Catalyst characterization: SEM, XRD, SBET. AOP comparison results for compound concentration and TOC. Repetitive experiments for catalyst reuse.	[43]
Orange II dye: 10^−4^ M. Processes: O_3_ 100 mL/min and 2 mg L^−1^ of ozone in influent gas. Processes: O_3_, O_3_ cat, O_2_/UV/cat, O_3_/UV/cat.	Bi_2_O_3_ and Au/Bi_2_O_3_ nanorods: 1 g L^−1^.	55 W Xe lamp with 20,200 Lux and a 320 nm cut-off filter. Wavelength > 320 nm.	A 500-mL capacity borosilicate glass photoreactor with walls covered with aluminum foil to avoid the release of radiation.	Microwave and hydrothermal methods of catalyst synthesis. Catalyst characterization: DRX, SEM, TEM, XPS, EDX, DRUV-visible. Dye absorbance and concentration. Role of photosensitization. Reusability studies. Byproduct identification.	[44]
2-Chlorophenol, 2,4-dichlorophenoxyacetic acid, bisphenol A, sodium dodecylbenzenesulfonate, sodium butylnaphthalenesulfonate, and benzyldodecyldimethyl- ammonium bromide surfactants at 0.5 and 0.1 mM in simulated wastewater. Processes: UV/O_3_, O_2_/TiO_2_/sunlight, and O_3_/TiO_2_/sunlight. 3 mg L^−1^ dissolved ozone.	Dispersed TiO_2_ P25 (0–2 g L^−1^) and TiO_2_-coated glass.	Solar light.	Pilot plant: three modules of 40 Pyrex glass tubes (inner diameter, 1.76 cm; length: 145 cm), each connected in series. Photoreactor volume: 42.3 L and total solar light harvest area of the three modules: 3.06 m^2^. Bubble ozone column connected in series with photoreactor modules. Solar cell for electric power to the photoreactor and ozonator.	Contaminants, chloride ion concentrations, and TOC followed vs. accumulated sunlight energy incident on the photoreactor per liter of the solution.	[45]
Dyes: Rhodamine B for UVA radiation and Methylene Blue for Visible radiation. At 10 mg L^−1^.	TiO_2_ and M-TiO_2_ catalysts. M = Ag^+^, Cu^2+^, Mn^2+^, Ce^3+^, Fe^3+^ and Zr^4+^ ions. Main catalyst used: Mn-TiO_2_ 0.25 g L^−1^ (0–7% Mn).	Lamp not given; full solar radiation wavelength (300 and above) and only visible light (λ > 400 nm).	Cylindrical Pyrex vessel surrounded by a cooling water jacket in a solar simulating box.	Catalyst characterization: DRX, SBET, DRUV-Vis. Absorbance of dyes solutions followed. Correlation between SBET and dye removal percentage.	[46]
Metoprolol (MTP) at 10 to 50 mg L^−1^ Processes: O_3_/Light, O_3_/Cat/Light, and all combinations.	1. Fe_3_O_4_/TiO_2_/AC 331 m^2^ g^−1^, 68% Anatase (0.38 g L^−1^) 2. P25 TiO_2_ (0.25 g L^−1^). Ozone gas inlet: 20 L h^−1^ and 6 mg L^−1^.	1500 W Xe lamp with limited radiation above 300 nm with filters. 550 W/m^2^.	Glass-made agitated tank provided with gas inlet, gas outlet, and liquid sampling ports.	Catalyst characterization: nitrogen adsorption, XRD, FTIR, SEM, EDX, SQUID magnetometer. Reusability and activity in five cycles. MTP Concentration. TOC followed.	[47]
Dichloroacetonitrile at 1 ppm. Ozone: 1–1.4 g L^−1^ h^−1^. Processes: UVsolar/TiO_2_, O_3_, O_3_/TiO_2_, UVsolar/O_3_, and UVsolar/TiO_2_/O_3_.	P25 TiO_2_ ozono dosage: 1–1.38 g L^−1^ h^−1^.	Three halide lamps (100, 250, and 400 W) and the Sun with similar light spectra from 300 to 800 nm.	1. Bench system: three halide lamps (100, 250, and 400 W), 60 cm at the top of three quartz tubes (40 cm × 2.7 cm). 2. CPC reactor (38° tilted) and cylindrical ozone reactor (1.1 m high, 10 cm internal diameter) in series. Turbulent regime.	Influence of different AOPs, pH (3, 6.5, and 10), W (4.6 to 33.8 W m^−2^), catalyst dosage 0.2–2.5 g L^−1^. Temperature: 10 to 40 °C.	[48]
Bisphenol A (BPA) and oxalic acid, 10 mg L^−1^. Ozone: 1 mL min^−1^ and 500 mgh^−1^. Processes: O_3_, UV-Vis/cat/O_2_, O_3_/UV-Vis/cat.	Graphitic carbon nitride (g-C_3_N_4_): Composed of numerous interconnected nanosheets, 0.5 g L^−1^.	High-pressure Xee long-arc lamp jacketed by a quartz thimble (GXZ500 W). Filter with Na_2_NO_2_ to cut λ < 400 nm.	One 1 L glass tubular photoreactor (8.5 × 40 cm).	Synthesis (from urea) and characterization TEM, FTIR, BET (67 m^2^ g^−1^), XRD, UV-Vis (2.7 eV, 450 nm max.). Rates for oxalic acid and BPA higher than sum of rates of single processes. *Tert*-butanol (TBA), triethanolamine (TEOA), and benzoquinone (BQ) act as the hydroxyl radical, hole and O_2_—scavenger.	[49]
Metoprolol, 50 ppm. Processes: O_3_/Light, O_3_/Cat/Light, and all combinations.	1. Fe_3_O_4_/TiO_2_/AC 331 m^2^ g^−1^, 68%. Anatase (0.38 g L^−1^) 2. P25 TiO_2_ (0.25 g L^−1^). Ozone gas inlet: 20 L h^−1^ and 6 mg L^−1^.	1500 W Xe lamp with limited radiation above 300 nm with filters. 550 Wm^−2^. 300–800 nm, 320–800 nm, and 390–800 nm.	Glass-made agitated tank provided with gas inlet, gas outlet, and liquid sampling ports.	Catalyst preparation and characterization (nitrogen adsorption, XRD, SEM, EDX, XPS and SQUID magnetometer). MTP concentration and TOC, dissolved O_3_, H_2_O_2_, acid intermediates.	[50]
Atenolol, Hydrochlorothiazide, Ofloxacin, and Trimethoprim in ultrapure water (10 mg L^−1^ doping) and WWSE (0.5 mg L^−1^ doping). Processes: O_3_, UVA-Vis, O_3_/cat, O_3_/UVA-Vis, O_2_/cat/UVA-Vis, O_3_/Cat/UVA-Vis.	P25 TiO_2_ 45 L h^−1^ and 20 mg L^−1^ for ozone in the inlet gas.	Solar light (visible + UVA) with: 35 ± 5 W m^−2^.	CPC: four tubes in series, 300–400 L h^−1^. Water flow rate for recirculation T = 18–30 °C.	ECs concentration, TOC, ecotoxicity (*Daphnia magna*), Phenolic compounds formed, BOD/COD. Ozone consumption.	[51]
Caffeine, metoprolol, and ibuprofen: 2 mg L^−1^ each in Municipal Wastewater MWW Processes: O_3_, UVA-Vis, O_3_/cat, O_3_/UVA-Vis, O_2_/cat/UVA-Vis, O_3_/Cat/UVA-Vis. 20 mg/L O_3_ and 20 L h^−1^.	WO_3_/TiO_2_ (from P25 and titanate nanotubes) 3.8%. WO_3_ and 0.5 g L^−1^.	1500 W Xe lamp with limited radiation restricted to wavelengths over 320 nm because of the presence of quartz, glass, and polyester cut-off filters with 550 W m^−2^.	0.5L semi-batch glass-made spherical reactor, provided with a gas inlet, a gas outlet, and a liquid sampling port in a commercial solar simulator chamber.	Catalyst characterization: ICP, N_2_ adsorption–desorption isotherms (SBET), XRD, TEM, Raman, XPS, and DRUV-Vis spectroscopy. Contaminant concentration and TOC followed.	[52]
Oxalic acid: 0.01 M (TOC = 240 ppm).	TiO_2_P25, Nb_2_O_5_, SnO_2_, WO_3_, Fe_2_O_3_, In_2_O_3_, and BiVO_4_: 2 g/L. O_3_: 14 mg L^−1^ and 0.45 g h^−1^.	300 W Xe lamp with an IR cut-off filter. Incident light was ca. 200 mW in the range of 360 to 470 nm. For only visible irradiation, another filter was used: λ > 410 nm.	Pyrex inner irradiation vessel placed in a thermostatic water bath.	SBET: 1.7 to 54.1 m^2^ g^−1^ TOC. Visible active properties of semiconductors (only WO_3_, Fe_2_O_3_, In_2_O_3_, and BiVO_4_). WO_3_: Best material for photocatalytic ozonation under visible light irradiation.	[53]
Ibuprofen: 10 mg L^−1^, and a mixture of acetaminophen, metoprolol, caffeine, hydrochlorothiazide, antipyrine, sulfamethoxazole, carbamazepine, ketorolac, diclofenac, and ibuprofen in an MWWT: 0.5 mg L^−1^ each Processes: adsorption, photolysis, O_3_, UVA-Vis, O_3_/cat, O_3_/UVA-Vis, O_2_/cat/UVA-Vis, O_3_/cat/UVA-Vis. Ozone processes: 10 mg L^−1^ and 20 L h^−1^ gas.	WO_3_: 0.25 g L^−1^.	1500 W air-cooled Xe arc lamp with emission restricted to visible light (λ > 390 nm) because of quartz, glass, and polyester cut-off filters. 550 Wm^−2^.	0.5-L glass-made spherical reactor in the chamber of a box simulator.	Preparation conditions: Calcination temperature and time. Characterization: TGA-DTA, XRD, N_2_ adsorption-desorption isotherms, pH_PZC_, XPS, and DRUV-Vis spectra. Contaminant concentrations and TOC followed. Mechanism, kinetic regime of ozonation.	[54]
*N*,*N*-diethyl-meta-toluamide DEET: 5 mg L^−1^ Processes: adsorption, photolysis, O_3_, UVA-Vis, O_3_/cat, O_3_/UVA-Vis, O_2_/cat/UVA-Vis, O_3_/Cat/UVA-Vis. Ozone processes: 10 mg L^−1^ and 15 L h^−1^ gas.	Commercial and homemade WO_3_ catalysts: 0.25 g L^−1^. Calcination temperature: 500 to 700 °C.	1500 W air-cooled Xe arc lamp with emission restricted to visible light (λ > 390 nm) because of quartz, glass, and polyester cut-off filters. 550 Wm^−2^.	0.5 L glass-made spherical Reactor in the chamber of a box simulator	Synthesis method. Catalyst characterization: XRD, Raman, N_2_ adsorption-desorption isotherms (SBET), SEM, XPS. Contaminant concentration and TOC followed. Oxalic, acetic, and formic acids were followed.	[55]
Acetaminophen, antipyrine, bisphenol A, caffeine, metoprolol, testosterone. Concentratoin: 1.5 and 2.9 mg L^−1^ (10^−5^ M each). Processes: Sun, O_3_, Fe(III), or P25 TiO_2_ combinations. Also, H_2_O_2_ for photo-Fenton.	Fe(III) (homog.: 2.79 mg L^−1^, pH 3), TiO_2_ (heterog.: 200 mg L^−1^, pH 7). In some cases: Fe(III)/H_2_O_2_ = 6.09 mass ratio. 13 mg L^−1^ ozone in gas.	The Sun. Average solar radiation: 40 W m^−2^.	Four borosilicate glass tube CPCs (29.4 × 75 cm). Collector surface 0.25 m^2^. Illumination volume: 1.8 L. Tilted 45° to the south. Parabolic anodized aluminum reflectors. Turbulent regime. Semi-batch mode.	Concentrations and TOC removal. Energy and ozone demands. Kinetic regimes. Kinetics.	[56]
Acetaminophen, antipyrine, bisphenol A, caffeine, metoprolol, testosterone in secondary WWSE effluent (BOD = 10, COD = 58.6; TOC = 20 mg L^−1^). Presence of anionic ions. Concentrations between 1.5 and 0.2 mg L^−1^. Processes: Sun, O_3_, Fe(III), or P25 TiO_2_ and their combinations. Also, H_2_O_2_ in some cases.	Fe(III) (homog.: 2.8 mg L^−1^, pH 3), TiO_2_ (heterog.: 200 mg L^−1^, pH 7). 13 mg L^−1^ ozone in gas.	The Sun. Average solar radiation: 40 Wm^−2^.	Four borosilicate glass tube CPCs (29.4 × 75 cm). Collector surface 0.25 m^2^. Illumination volume: 1.8 L. Tilted 45° to the south. Parabolic anodized aluminum reflectors. Turbulent regime. Semi-batch mode. Reaction time: 5 h (+30 min, previous for adsorption).	Concentrations and TOC removal. Also, ions and total phenol concentration. *Daphnia magna* ecotoxicity measurements. Measurements of HO· radicals concentration with *p*-chlorobenzoic acid as a probe compound. Biodegradability as BOD/COD. Economic considerations.	[57]
Diuron, *o*-phenylphenol, 2-methyl-4-chlorophenoxyaceticacid (MCPA), and *tert*-buthylazine (5 mg L^−1^ each). Processes: O_3_, UVA-Vis, O_3_/cat, O_3_/UVA-Vis, O_2_/cat/UVA-Vis, O_3_/cat/UVA-Vis.	TiO_2_ and 0.5–0.8 wt. % B-TiO_2_ 0.33 g L^−1^. Ozone in the inlet gas: 5 mg L^−1^ and 10 L h^−1^.	1000 W Xe lamp. Incident radiation flux: 8.96 × 10^−4^ Einstein min^−1^. Radiation intensity: 500 W m^−2^.	Glass-made agitated tank provided with gas inlet, gas outlet, and liquid sampling ports in a solar simulator box.	Synthesis and characterization: ICP-OES, N_2_ adsorption–desorption, XRD, XPS, and DRUV-Vis spectroscopy (3.01 and 3.03 eV band gap) Compound concentration. TOC, dissolved ozone, and H_2_O_2_ concentrations. B leached.	[58]
Acetaminophen (ACM), antipyrine (ANT), caffeine (CAF), ketorolac (KET), metoprolol, sulfamethoxazole (SFX), carbamazepine (CARB), hydrochlorothiazide (HCT), and diclofenac (DIC). In WWPE doped: 200 μg L^−1^ each. Processes: solar photocatalysis with O_3_/TiO_2_, solar photo-Fenton, or ozonation.	Three catalysts: 1. pH 3 with 2.8 mg L^−1^ Fe(III). 2. 150 mg L^−1^Fe_3_O_4_. 3. Natural pH with 250 mg L^−1^ P25 TiO_2_. Ozone in the inlet gas: 33.6 L h^−1^ and 13 mg L^−1^.	Radiation source: Sun.	Aerobic tank: HRT of 7 h, biomass sludge aged 5–6 days. MLVSS.MLSS^−1^: 0.8. Oxygen: 2–4 mg L^−1^. Reactor Volume: 5 L (1.8 L of irradiated volume) compound. Parabolic collector. Four borosilicate glass tube CPCs (29.4 × 75 cm).	Aerobic degradation followed by AOP, solar photocatalysis. ECs concentration, TOC, COD, ecotoxicty. Accumulated UV-vis energy calculated.	[59]
Bisphenol A and oxalic acid (OA), 10 mg L^−1^ at 1 mL min^−1^ and 500 mgh^−1^.	Graphitic carbon nitride: g-C_3_N_4_ composed of numerous interconnected nanosheets. Concentration: 0.5 g L^−1^	High-pressure Xe long-arc lamp, jacketed by a quartz thimble (GXZ500 W). Filter with Na_2_NO_2_ to cut λ < 400 nm.	1 L glass tubular photoreactor (8.5 × 40 cm).	Synthesis (from urea) and characterization (TEM, FTIR, BET (67 m^2^/g), XRD, UV-Vis (2.7 eV, 450 nm max.). Rates for oxalic acid and BPA higher than sum of single processes: O_3_ and UV-Vis/cat/O_2_. *Tert*-butanol, (TBA), triethanolamine (TEOA), and benzoquinone (BQ) as HO·, hole and O_2_—scavengers.	[60]
Oxalic acid, 0.11 mM.	gC_3_N_4_-reduced graphene oxide (rGOxide) 0.2 g/L. Ozone: 75 mgh^−1^.	As in Reference [56].	As in Reference [56].	Catalyst characterization. 2% rGO leads to best results for OA removal. Catalyst activity and stability. Basic mechanism.	[61]
Phenol in water and urban wastewater: 50 mg/L (DOC: 20 mg L^−1^, COD: 40 mg L^−1^, pH 7.4) Processes: Ozonation, photocatalysis, and photocatalytic ozonation	Ag, Cu, Fe on TiO_2_: 0.5 g L^−1^ Ozone: 20.83 mg L^−1^ min^−1^	Heraeus TQ 150 W immersion medium-pressure Hg lamp 70 mW cm^2^. The lamp emission spectrum has main peaks at 253.7, 313, and 366 nm in the UV range and 436, 546, and 578 nm in the visible range. For solar runs: 37.6 mW cm^−2^.	For UV runs: cylindrical quartz photochemical reactor (0.7 L) wrapped in aluminum foil. For solar runs: glass tubular reactor (1 m in length and 0.04 m in diameter) and a parabolic solar collector.	Best catalyst: Fe-TiO_2_ which presents the highest BET area and higher λ visible absorption (530 nm). Phenol concentration. COD, Langmuir kinetics applied simplified to pseudo first order kinetics. Synergic index, pseudo first rate constants calculated.	[62]
*t*-Butilazina: 5 mg L^−1^. Processes: Adsorption onto AC and multi-walled carbon nanotube (MWCNT), UV photolysis, UV/H_2_O_2_, single ozonation, O_3_/H_2_O_2_, catalytic ozonation (AC, MWCNT and TiO_2_ as catalysts) and some solar driven processes such as photo ozonation, TiO_2_-photocatalytic oxidation, and TiO_2_-photocatalytic ozonation.	AC (DARCO^®^, 12–20 mesh) and MWCNTs (purity > 95%) carbon, P25 TiO_2_, and other TiO_2_ prepared and TiO_2_-MWCNT. Ozone: 10 mg L^−1^, 20 L h^−1^.	Low-pressure Hg lamp with emission at 254 nm (Heraeus, model TNN15/32). Average fluence rate: 0.6 W–1 (2.9 mW cm^−2^). For UVA-visible: 1000 W Xe lamp (300–800 nm). 581 W m^−2^ (62 Wm^−2^ UV-A irradiance.	For UVC photolysis and UV/H_2_O_2_: photoreactor provided with a central quartz well. For O_3_ and O_3_/cat runs: 400 mL semi-batch spherical Pyrex-made reactor provided with magnetic agitation. For photoprocesses: The same reactor as in ozone processes inside a Suntest solar simulator equipment	Characterization: XRD, SBET, TEM, FTIR. Isotherm and different AOPs and adsorption kinetics TBA concentrations and intermediates by HPLC-qTOF.	[63]
*N*,*N*-diethyl-meta-toluamide (DEET): 5 mg L^−1^. Processes: Different AOPs and adsorption.	Two CeO_2_ catalysts: nanorod and nanocubes. Hydrothermal method. Concentration: 0.25 g L^−1^. Ozone: 10 mg L^−1^. 15 L h^−1^.	1500 W Xe lamp: 550 W m^−2^ and 300 to 800 nm or 400 to 800 nm with filter.	Semi-batch borosilicate glass-made round flask in a Suntest CPS solar simulator.	Catalyst preparation and characterization: XRD, XPS, SBET, DR UV-Vis. DEET concentration, TOC, O_3_ dis. H_2_O_2_, short chain carboxylic acids. Pseudo first order kinetics.	[64]
*N*,*N*-diethyl-meta-toluamide (DEET): 15 mg L^−1^. Ozone processes: 10 mg L^−1^, 15 L h^−1^ gas flow rate.	Monoclinic WO_3_ calcined at 600 °C (see Mena et al., 2015): 0.25 g L^−1^.	1500 W air-cooled Xe arc lamp with emission restricted to visible light (λ > 390 nm) because of quartz, glass, and polyester cut-off filters. 550 W m^−2^.	0.5 L glass-made spherical reactor in the chamber of a box simulator.	HPLC-qTOF identification of intermediates. Mechanism and kinetics based on TOC removal. Scavengers used: t-butanol and oxalate. Arrhenius equation determined for DEET-O_3_ reaction.	[65]

^a^ COD: Chemical Oxygen Demand. AC: Activated Carbon. FTIR: Fourier Transform Infrared. SEM: Scanning electron microscopy. XRD: X-ray diffraction. SBET: Surface area from Brunauer-Emmer-Teller isotherm. TEM: Transmission electron microscopy, XPS: X-ray photoelectron spectroscopy. DRUV-Vis: Diffuse reflectance UV-Visible spectroscopy, EDX: Energy dispersive X-ray spectroscopy, SQUID: Superconducting quantum interference device, WWSE: Wastewater secondary effluent. BOD: Biological Oxygen Demand. ECs: Emerging contaminants. WWPM: Wastewater primary effluent. ICP-OES: Inductively coupled plasma-optical emission spectroscopy. HRT: Hydraulic residence time. HPLC-qTOF: High performance liquid chromatography coupled with quadrupole time-of-flight mass spectrometry. TGA-DTA: Thermogravimetric and differential thermal analysis.
